# Fruits and Vegetables in the Management of Underlying Conditions for COVID-19 High-Risk Groups

**DOI:** 10.3390/foods10020389

**Published:** 2021-02-10

**Authors:** Nora A. Moreb, Ahmed Albandary, Swarna Jaiswal, Amit K. Jaiswal

**Affiliations:** 1 School of Food Science and Environmental Health, College of Sciences and Health, Technological University Dublin—City Campus, Grangegorman, 7 Dublin, Ireland; noramoreb@gmail.com (N.A.M.); a.albandary1990@gmail.com (A.A.); swarna.jaiswal@TUDublin.ie (S.J.); 2 Environmental Sustainability and Health Institute (ESHI), Technological University Dublin—City Campus, Grangegorman, 7 Dublin, Ireland

**Keywords:** immune system, COVID-19, virus, fruits and vegetables, underlying conditions, health benefits, micronutrients, food safety

## Abstract

SARS-CoV-2 or COVID-19 is a novel coronavirus, which is the cause of the current pandemic with 107,411,561 infections and 2,351,195 death worldwide so far. There are multiple symptoms that are linked with the infection of COVID-19 such as coughing, shortness of breath, congestion together with fatigue, fever, loss of taste or smell, headaches, diarrhea, vomiting, and loss of appetite. The lack of or early stage of development of a cure for COVID-19 illness, there is need for insuring the best possible position of health to be able to fight the virus naturally through a robust immune system to limit severe complication. In this article, we have discussed the role of fruits and vegetables consumption to boost the immune system and major emphasis has been given to high risk group. We have taken into consideration a number of underlying conditions such as people with cardiovascular diseases, obesity, diabetes, chronic obstructive pulmonary disease, chronic kidney disease, hemoglobin disorder such as sickle cell disease, weakened immune system due to organ transplant. Furthermore, factors to improve the immune system, risks associated with quarantine and lifestyle and food handling during COVID-19 has been discussed.

## 1. Introduction

COVID-19 is a novel coronavirus that caused the global pandemic of 2020 [1]. Corona viruses are a class of viruses that get their name from the crown like spikes (corona) that are observed on the surface of the virus particles and were identified in the 1960s. This class of viruses includes many common viruses like 229E and NL63. which commonly infect humans, causing mild cold-like symptoms, but they can also infect animals and evolve into new or novel viruses such as MERS (Middle East Respiratory Syndrome) and SARS-CoV (Severe Acute Respiratory Syndrome) [2]. COVID-19 is the latest illness caused by a novel coronavirus and was designated COVID-19 by WHO (World Health Organization) on 11th February 2020. The name is an abbreviation ‘CO’ is for ‘Corona’, ‘VI’ stands for ‘virus’, while ‘D’ stands for disease and ‘19′ for the year 2019 when the virus was discovered [3].

There are multiple symptoms that may appear in people infected by COVID-19, which can include respiratory symptoms such as coughing, shortness of breath, and congestion. COVID-19 can also include other non-respiratory symptoms such as fatigue, fever, loss of taste or smell, headaches, diarrhea, vomiting, and loss of appetite, as well as, more severe symptoms such as bluish face or lips, confusion, and inability to wake or stay awake [4].

In addition to the various symptoms associated with COVID-19, people infected will also experience the illness at various degrees of severity; people with pre-existing conditions may increase the likelihood of severe illness as a result of the infection. These conditions include, chronic kidney disease, chronic obstructive pulmonary disease, diabetes, and haemoglobin disorders such as sickle cell disease and more. People with pre-existing conditions must take extra precautionary steps to limit their risk of infection due to their higher risk of developing severe symptoms or death [5].

Due to the fact that COVID-19 is a novel virus, the lack of or early stage of development of a cure for COVID-19 illness and the vaccine has been just developed and not proven yet to cure the illness while the antiviral medication in the early stage of development [6]. The lack of a cure for this illness has highlighted the need to insure the best possible position of health to be able to fight the virus naturally through a robust immune system to limit severe complication and long-term complications.

The aim of this article is to provide information on the role of fruits and vegetables consumption to boost the immune system and major emphasis has been given to high-risk groups such as people with underlying conditions for example people with cardiovascular diseases, obesity, diabetes, chronic obstructive pulmonary disease, chronic kidney disease, hemoglobin disorder such as sickle cell disease, weakened immune system. Furthermore, factors to improve the immune system, risks associated with quarantine and lifestyle and food handling during COVID-19 has been discussed.

## 2. Factors to Improve the Immune System

The main role of immune system is to maintain the body’s health by maintaining the coherence of the cells and tissues safely. The framework of the immune system has two strategies that are innate immunity and adaptive. Both have the same goal: to protect the body. First, the subsystem works directly after infiltration of microorganisms or when danger signal appears [7]. It is considered as the first line of defense in anticipation of and preparation for protecting against harmful substances by the external barriers of our body such as skin. The adaptive immunity is activated after exposure to special germs by making antibodies to fight them [8]. This strategy is slower compared with the innate immunity [7]. The immune system has the ability to fight cold and other type of the infection to protect the human’s health. Despite the importance of the immune system in protecting health, it could fail in adequately protecting the body when it is weak. Therefore, it is very important to support the immune system by following a good lifestyle [9]. As there are many factors that could affect the strength of the immune system such as sleep, travel, diet and other [10].

### 2.1. Importance of Exercise

To slow the spread of COVID19, numerous health authorities around the world have recommended the public to stay at home, which impacted day to day life activities such as physical activity. Sedentary lifestyle could increase the risk of obesity, hypertension, and type 2 diabetes mellitus [11]. Increasing consumption of food and reduction of the physical activities may lead to weight gain [12].

The effects of exercise on efficiency of the is dependent on many factors that include the type of exercise, level of performance, and the period of training [10]. Maintaining a healthy level of physical activity is important to the human physical and mental health [11]. According to Harvard Health Publishing, regular exercise is essential for a healthy life [13]. It can contribute in maintaining cardiovascular health, can help control body weight, and protect the individual against many diseases. Moreover, exercise can maintain an effective immunity response.

Exercise can be divided into three categories, primarily: aerobic, anaerobic and agility training, all of which have a huge benefit to the human body [11]. It has been found that the strength of the immune system has a significant role against COVID-19. It helps in increasing the aerobic capacity, which may lead to improved immunity by increasing the level and function of T-lymphocytes, neutrophils, macrophages, and monocytes, which are considered the essential mechanisms in fighting against infections [14]. Subsequently, it has been recommended to exercise regularly for 10 to 30 min a day [14]. Researchers have observed that regular exercise increases blood flow which leads to better distribution of immune system cells (white blood cells around the body to protect against diseases) [15].

### 2.2. Hydration (Drinking Water)

The human body consists roughly 60% water. Although it is a general guideline, health experts recommend to drink at least 8 cups of water every day to avoid dehydration [16]. Dehydration can cause many health issues that include headaches, skin problems, muscle cramps, low blood pressure and a rapid heart rate [17]. Even mild dehydration could cause hypertension [18] and subsequently could affect the immune system [19].

Additionally, water can aid in maintaining every system in the body to function properly. Hydration also helps in transporting nutrients and oxygen to cells, prevent constipation, maintain blood pressure, protect organs and tissues, regulate body temperature, and more [20]. Drinking water can boost the adaptive immune response by removing toxins from the body through the kidneys and sweat glands, which may increase the immune function. Moreover, it increases the production of lymph fluid, which is responsible to flow around the body to collect bacteria and waste and transport them to lymph nodes where lymphocytes destroy them. This lead to promote the immune function [21]. Together with water, numerous fruits and vegetables and some beverages, which are rich in water and can contribute to keep the body’s hydration, such as cucumber 95%, zucchini 94%, tomatoes 94%, watermelon 92%, cauliflower 92%, cabbage 92%, strawberries 91, cantaloupe 90%, peaches 89%, oranges 88%, grapefruit 88% and skim milk 91% [17].

### 2.3. Healthy Eating Habits to Help the Immune System

The immune system protects the body against diseases, and can be enhanced with a healthy diet [20]. Many studies have found that a healthy diet can protect against diseases including those related to oxidative and inflammatory processes. For example, citrus fruits are high in vitamins, flavonoids, antioxidants phytochemicals, minerals, and other nutrients, and play an important role in enhancing immunity and preventing many diseases such as cardiovascular diseases and metabolic diseases including obesity, diabetes mellitus and some types of cancer [22]. Additionally, nutrients in citruses have many physiological properties that are considered as anticancer, antiviral, anti-microbial and anti-inflammatory activities [23,24].

Another source of food that can boost the immunity is broccoli, as it contains vitamin C and sulforaphane (which is anticarcinogenic) [25]. Garlic as well in another good food source, because it contains sulfur-containing compounds, such as allicin, which many studied showed the effectiveness of garlic against many viruses, such as influenza A and B, cytomegalovirus, rhinovirus, HIV, herpes simplex virus 1, herpes simplex virus 2, viral pneumonia, and rotavirus [26]. In addition, fruits such as cranberries, blueberries, prunes, red delicious apples, granny smith apples, gala apples, sweet cherries, plums, and blackberries also contain a significant level of antioxidants [25]. Furthermore, vegetables that contain a high level of antioxidants are spinach, kale, red and green chili peppers, red cabbage, red beets, black and green olives, okra, beans, artichokes and russet potatoes [25].

There are many factors that should be considered in aiding medical condition(s) to help maintain the immune system. One of these factors is adapting a healthy lifestyle that advocates for an adequate consumption of various fruits and vegetables (FV) depending on the condition to aid with the overall boosting of the immune system, which will be discussed in the next section.

## 3. Fruits and Vegetables and COVID19 High Risk Groups

All individuals are at risk of getting COVID-19, however, individuals who have some underlying conditions, such as: serious cardiovascular or cerebrovascular diseases, obesity, diabetes type 2 mellitus, chronic obstructive pulmonary disease (COPD), chronic kidney disease, hemoglobin disorder such as sickle cell disease, immunocompromised state (weakened immune system) from solid organ transplant and cancer are at higher risk and are more likely to become severely ill regardless of their age than others who are healthy. Moreover, individuals that have more than one underlying medical condition been at higher risk, and the risk increases with the increase of underlying medical conditions. The severity of their illness from COVID-19 could require hospitalization, intensive care, ventilators in order to breath or may even lead to death. Therefore, it is especially important to keep their conditions under control [2,5].

Generally, a diet rich in fruits and vegetables is regarded as healthy and beneficial in a number of conditions (please refer to later sections). It is recommended by the dietary guidelines to have at least five servings of FV a day. Table 1 presents fruits and vegetables and its associated beneficial nutrients for reference purpose.

### 3.1. Serious Cardiovascular or Cerebrovascular Diseases

According to Dr. Mitchell Elkind, professor of neurology and epidemiology at Columbia University in New York City, cardiovascular diseases (CVD) does not seem to increase the risk of contraction of COVID-19, rather it increases the severity of COVID-19 complications and infection Examples of serious cardiovascular diseases that can compromise getting severely ill from COVID-19 includes: heart failure, coronary artery disease, cardiomyopathies, and pulmonary hypertension. There are other conditions that may increase the risk of getting seriously ill with COVID-19 such as: high blood pressure, stroke and more [2,53,54].

According to the CDC data, 1 out of 3 patients have CVD as an underlying condition; thus, it is the “most common” underlying medical condition with COVID-19 patients. Also, CVD patients are considered high risk patients because they are six times more likely to have severe COVID-19 symptoms that requires hospitalization, and the likelihood of death is higher by 12 times [54].

CVD is often associated with severe symptoms of COVID-19 and higher risks of fatality. “COVID-19 can have both primary (arrhythmias, myocardial infarction, and myocarditis) and secondary (myocardial injury/biomarker elevation and heart failure) cardiac involvement. In severe cases, profound circulatory failure can result” [55]. Therefore, the CDC recommends having a continuous heart-healthy lifestyle of exercise (such as: having 75 min of moderate exercise and muscle training twice a week) and healthy foods (such as: high intake of fruits and vegetables, whole grains, and substitute meats and dairy for lean meat and low-fat dairy, and decrease saturated fats, added sugar and salt), which is so important and makes a huge difference over time [56]. Based on a fifty-year follow-up study done in seven countries, results showed that a higher intake saturated fats and sodium increases the rates of CVD and mortality rate. Moreover, CVD was reduced with a healthy diet rich in “healthful complex carbohydrates” such as starchy vegetables and various nutrients and minerals have been shown to help with CVD and its symptoms [56,57,58,59].

Additionally, cardiovascular (CV) events were reduced when decreasing triglyceride and low-density lipoprotein (LDL). In another study done on 19,914 adults of the ages of 20 and above showed that the association of higher intake of FV had a lower probability risk of high triglycerides, hypertension and CV events, and diets low in FV were responsible for at least half the deaths caused by CVD in the United States (US). When comparing patients with a deficiency in micronutrients to those who do not have a deficiency, patients with a deficiency had at least double the CV events; thus, micronutrients from FV are essential to heart health and CVD. Additionally, various studies showed a strong correlation between the Mediterranean diet which contains lots of FV and <7 glasses of fruit wine per a week on reducing CVD and total all-cause mortality [33,56,57,60,61,62].

It is recommended by the dietary guidelines to have at least five servings of FV a day which is equivalent to “about two and half cups” which has a slight reduction of CVD. Moreover, increasing consumption of FV to 10 servings (about five cups) reduces CVD by 28% and premature mortality by 31%. However, generally increasing intake is beneficial, but intake of certain types and preparation methods of FV should be limited as it could have an adverse effect on CVD, such as: coconut, fruits or vegetables that are fried, breaded or dipped in creamy sauces, and canned and frozen fruits that contain added sugars and salt [63,64,65].

#### Beneficial Nutrients and Minerals

Fiber is an indigestible carbohydrate which is beneficial to people suffering from CVD [66,67]. Potassium is also beneficial for people with CVD; it has also been found to help regulate blood pressure and lower the risk of stroke [58,66,68,69]. Antioxidants including all types of flavonoids and phytonutrients; which have been observed to have antioxidant, antimicrobial, pharmaceutical and biological activities in the prevention of common diseases and illnesses, when consumed from fresh FV [6,27,32,70]. Omega-3 polyunsaturated fatty acids has also been found to be beneficial for people with CVD [57]. Magnesium has been found to decrease the risk of CVD events. It has also been found to improve heart function and may reduce the risk of complications such as arrhythmia and heart failure [71,72]. Vitamin B is also beneficial for people suffering from CVD as B6 & B12 have been found to be associated with a lower risk of coronary heart disease while B9 (known as folate) has been associated with lower risk of stroke [73,74].

### 3.2. Obesity

According to WHO, obesity is a global epidemic; an estimated 52% of adults globally are overweight or obese. Obesity increases the risk of other medical conditions such as type 2 diabetes, CVD, hypertension, morbidity, and a shorter lifespan. Obesity is categorized by having a Body Mass Index (BMI) that is >30; and it is mainly caused by the “modern-day lifestyle” of making poor diet decisions combined with a high-calorie diet, high carbohydrate intake, low intake of FV, and low activity [64,75,76,77,78,79].

Various observations have shown that all individuals at all ages who are obese are at a higher risk of potential complications and consequently high severity illness from COVID-19. Moreover, people who are older and obese are at even higher risk. Additionally, individuals with obesity might also suffer from other complications that makes fighting COVID-19 even harder, such as sleep apnea that could increase pulmonary hypertension, or have a larger body mass that makes it difficult with the hospital setting and intubation [5,80,81].

Based on a global pooled analysis study, findings show that individuals who are obese have 46% higher risk of being positive for COVID-19. This could be due to the Angiotensin-Converting Enzyme 2 (ACE2), which is a receptor protein that binds with COVID-19 accelerating its entry into the cell. ACE2 is found in high concentrations of lipids/fats (“fat-cells”). It is also found in the cells of the brain, intestinal, heart, lungs, mouth, testes, kidney, and liver, therefore, high expression of ACE2 could increase infection and mortality, and the virus degrades and inactivates the receptor. Individuals that contract COVID-19 in conjunction with the underlying condition of obesity are also 113% more likely to get complications that requires hospitalization, 74% more likely to get admitted into the ICU, and have a mortality increase of 48% when compared to healthy individuals that contract COVID-19 [80,81,82].

Furthermore, individuals with a high BMI (example: 35 and higher), have most of their weight in the abdominal area, which in return interferes and restricts the mechanism of the diaphragm, making it harder to breathe. This is especially true with respiratory diseases such as COVID-19, where doctors are noticing that the membranes between the “lung airways sacs” and blood vessels are leaking, thus, making it challenging to get oxygen into the blood stream, which in turn requires the diaphragm to work more efficiently and harder while obesity restricts it. Obesity also increases inflammation in the body due to the metabolism of fat, which results in activating the immune system response, and when obesity’s symptoms are combined with the novel virus of COVID-19, the immune response is increased making the immune system hyper-activated, which in turn increases the risk and severity of complications [80].

Therefore, overcoming obesity is highly recommended, and weight loss is needed. There are many examples of healthful diets such as are the Mediterranean diet, plant-based diets and more; although, there is no one universal diet that is recommended. Nevertheless, the WHO recommends a daily intake of 400 g (a minimum of 5 servings) of FV to aid with weight-loss and reduce the risk of obesity. Moreover, the most recommended healthful diet is one that is particularly rich in a variety of FV [61,62,70,75,76,78,79,81,83,84].

It is essential to have an adequate intake of whole FV, as it benefits several mechanisms in aiding weight loss and reducing the eating rate, due to the fact that it is the main source of dietary fiber, so it is satisfying when consumed in adequate levels, and it is low in fat, glycemic load, and energy making it a low caloric density food. High intake of FV also increases essential nutrients such as: vitamins, minerals, omega-3 polyunsaturated fatty acids, and they are high in antioxidants & bioactive compounds, like flavonoids & sterols. Examples of beneficial FV includes: yellow/orange vegetables, ginger, green peas and more [61,70,76,78,79,83,85].

Generally, a diet rich in fruits and vegetables are regarded as healthy and beneficial to overcoming obesity; however, it is important for everyone to be cautious in in their choices, limiting intake of certain preparation methods of FV such as: fruits or vegetables that are fried, breaded or dipped in creamy sauces, and fruit juices, because although simple sugars in fruits (such as: fructose and glucose) are natural, nevertheless, it could easily compile into large quantities of simple sugars, which can be unhealthy when consumed in large quantities and lead to central weight gain (fat/lipid deposits). Also, limiting the intake of processed and ultra-processed foods/drinks; based on various consistent studies, there is a clear link between ultra-processed foods (such as: ready-to-eat foods/drinks and more), which tends to be calorie (energy) dense, high in saturated fats, sodium, and sugar and adverse health effects including obesity. Moreover, the diet/lifestyle should also consistently be low in sodium, sugar such as fructose, saturated fats, high-glycemic index foods and starchy vegetables like tubers, which includes potatoes and cassava [65,76,78,79,81,83].

### 3.3. Diabetes

Type 2 diabetes is a chronic condition that most often develops in adulthood to late adulthood, however, it is becoming more common in children and teens. The disease primarily affects blood glucose by allowing its levels to rise to a harmful extent. Diabetics are resistant to insulin, which is a hormone produced by the pancreas, when blood glucose rises, the pancreas produces insulin to signal cells to absorb it. When cells are resistant to insulin, they stop absorbing glucose efficiently which allows the glucose levels to rise triggering the pancreas to produce more insulin which in turn increases resistance over time, so it is a degenerative illness [86].

Type 2 diabetes, and possibly type 1, increases an individual’s risk of severe illness if infected with COVID-19. Roughly, 20–50% of global COVID-19 patients have diabetes so it is one of the most common underlying conditions affecting patients. Based on a study that included 33 global studies of 160,003 patients, found that individuals with diabetes have two-folds of an increased risk of severity, and mortality from COVID-19. Another study showed that COVID-19 diabetic patients had an increased risk of severe complications, such as respiratory distress syndrome, organ failure, Diabetic Ketoacidosis (DKA), and death is 50% higher. This chronic condition has been listed as a comorbidity for all three coronavirus infections and has been known to increase the risk of acute respiratory syndrome and multi organ failure [2,82,87,88].

Diabetics’ symptoms are closely related to high blood glucose concentration and include: frequent urination and thirst or hunger, changes in weight, high risk of infections, dehydration, which in extreme cases can develop into hyperosmolar syndrome, it is important to maintain healthy activity levels as well as eat a diet rich in fruits and vegetables in order to prevent diabetes or to manage its symptoms [86]. The CDC recommends that people suffering from diabetes should prevent dehydration by drinking at least 4–6 ounces of fluids every half hour and to consume roughly 50 g of carbohydrates every 4 h from small amounts of sweetened drinks if eating is not an option or food does not provide enough carbohydrates [89]. The CDC recommends that people suffering from diabetes should prevent dehydration by drinking at least 4–6 ounces of fluids every half-hour and to consume roughly 50 g of carbohydrates every 4 h from small amounts of sweetened drinks if eating is not an option or food doesn’t provide enough carbohydrates [89].

Another key symptom for diabetics to manage is ketoacidosis. Ketoacidosis occurs when ketones, which are formed in the liver from free fatty acids, reach abnormally high concentrations in the blood. One recent study showed that COVID-19 patients with diabetes were more susceptible to this condition and spent longer times in the hospital and had a higher mortality rate. The study also showed that non-diabetics with COVID-19 also experienced ketoacidosis, suggesting a link between the disease and the way fat is broken down which further stresses the need for increased focus on diet in regard to COVID-19 [87].

Diabetic ketoacidosis (DKA) can also make it more difficult to manage fluid and electrolyte intake which in turn leads to dehydration and can also make it more difficult to treat sepsis, which is a common complication experienced by COVID-19 patients, so it is important to check ketone levels if blood glucose readings are high (above 240 mg/dL in two consecutive readings). It is important to note that the virus may thrive in patients with high blood glucose, so it is important to monitor glucose levels [90,91]. It is important to note that the virus may thrive in patients with high blood glucose, so it is important to monitor glucose levels [90,91].

A study focused on the global burden of diabetes showed that people who ate sufficient quantities of fruits and vegetables had a better chance at avoiding some of the complications associated with diabetes especially in relation to the cardiovascular system and obesity as well as an overall reduction of a fatal outcome. Diabetes, being an epidemic disease that already has a heavy burden on human health, has received plenty of attention from nutritionists and although no one diet is considered the best diet, patterns have emerged that suggest that diets with a wide variety of fruits and vegetables is beneficial in managing many of the symptoms and complications associated with type 2 diabetes as well as other complications that cause insulin resistance such as polycystic ovary syndrome [61,64,83].

As diabetes is a condition that is closely related to diet, it is important to have a healthy diet and lifestyle in order in order to manage this condition and reduce the risk of serious illness if infected with COVID-19. A diabetic should pay close attention to blood glucose and keep carbohydrates in mind when making dietary choices. Some recommendations are: to have about two to three servings of fruits, eat plenty of vegetables including a variety of leafy green vegetables and other foods that also have a low glycemic index like wheat, reduce intake of fried foods, reduce intake of foods high in carbohydrates, sugar, and fat, and try home exercises or other safe activities to keep an active lifestyle [91].

#### Beneficial Nutrients and Minerals

Adequate supply of chromium has been shown to help control blood sugar levels and is important in using glucose effectively. It is important to note that too much chromium may cause kidney damage and skin reactions [92]. Magnesium is used by the body to process glucose and can be helpful in preventing diabetes so it can be beneficial to people who are at risk of developing this condition [92]. Studies have shown that vitamin D deficiency is associated with an increased risk of type 2 diabetes and other metabolic disorders. It does not appear to prevent the disease, but it should be incorporated in a healthy diet for its anti-inflammatory properties as well as its role in a healthy immune system and it has been shown to have a beneficial effect in reducing the risk of cardiovascular complications in diabetic patients [92,93]. Studies have shown that an increase in intake of vegetables high in vitamin A helped reduce intra-abdominal fat making it important for weight management [94]. Studies have also shown that this micronutrient can help prevent diabetes, reduce insulin resistance, and help with glucose level management [95].

Glucose can compete with vitamin C in cell absorption, which can lead to a deficiency of this vital antioxidant. Adequate supply of vitamin C and proper glucose control are essential [96]. Vitamin E, a strong antioxidant that has been shown to reduce the risk of long-term complications associated with diabetes [97]. Zinc has been shown to have an effective role in protecting against long-term complications from diabetes especially for the liver [98]. Calcium has been shown to decrease the risk of long-term complications associated with diabetes [99]. Selenium has been found that it has a protective effect for the pancreas and that it can help reduce blood glucose [100]; another study found that vanadium can help manage glucose levels and reduce inflammation biomarkers [101].

As these nutrients and minerals can help manage diabetes symptoms and complications, it is likely that having them incorporated into a diabetic’s diet should reduce the chances of serious illness or death if infected with COVID-19, as well as their overall health benefits managing the symptoms of diabetes.

### 3.4. Chronic Obstructive Pulmonary Disease (COPD)

COPD is a group of diseases that cause the obstruction of the airways in the respiratory system making it harder to breath. While COVID-19 can cause breathing problems in patients, having COPD as a pre-existing condition can further exacerbate the symptom greatly increasing the risk of severe illness and even death [102].

SARS-CoV 2 (the virus that causes COVID-19) attacks the respiratory system so having a chronic respiratory disease increases the risk of severe illness. A recent report released by the CDC showed that one-third (34.6%) of COVID-19 hospitalized patients suffered from a pre-existing lung disease [103]. Since chronic lung diseases such COPD were observed in a significant percentage of hospitalizations it is important to address the issues related to these illnesses proactively to increase the chances of recovery if infected.

According to the American Lung Association, nutrition plays an important role in managing COPD and fresh fruits and vegetables are especially beneficial for COPD patients [103]. A recent study by the Italian Institute of Clinical Physiology also found that diets high in fruits and vegetables and healthy fats were beneficial for people suffering from COPD by reducing the oxidative stress and inflammation, which improved lung function [104]. A study in the UK has also found that people with COPD are prone to malnutrition and that approximately 22% of outpatients in the UK with COPD suffered from malnutrition [105]. Malnutrition has been a well-documented cause of a weak immune system and increased risk of infection [106], so malnutrition especially in older COPD patients is something that must be since they are more prone to micronutrient deficiencies that are vital to a healthy immune system.

#### Some Key Nutrients

Iron is an essential mineral for our body as it is used by our blood cells to transfer oxygen from our lungs to cells in our body. Due to the role of iron in transporting oxygen throughout our body it is important for people who suffer from COPD to ensure that they prevent deficiencies and maintain a healthy intake of iron in their diet. Iron deficiency has been shown to cause difficulty breathing, low exercise tolerance, and an increased risk of COPD exacerbations. Iron is also important for a healthy immune system our bodies use it for our T-cells as well as intercellular pathogen elimination [107].

Ascorbic acid (Vitamin C), is a water-soluble antioxidant that the body uses for detoxification and as means of fighting infections. As an effective antioxidant, Vitamin C has been known to reduce the oxidative stress in the body as well as reduce inflammation [108]. Vitamin C has also been shown to protect the lungs epithelial cells from damage due to oxidative stress. These benefits combined make Vitamin C a very desirable compound for COPD patients especially during the COVID-19 pandemic.

Magnesium is an essential mineral found in many dietary sources and is involved in more than 300 enzyme systems in the human body playing an important role in various bodily function from muscle and nerve function and energy production, to synthesis of the antioxidant glutathione as well as having an important role in transporting calcium and potassium across cell membranes. Higher serum magnesium concentrations are associated with stable COPD symptoms while lower concentration is associated with patients that have exacerbated COPD, which highlights its significance concerning COPD management [109]. Moreover, magnesium deficiency has been found to be a high risk factor for Acute Exacerbated COPD [110] so ensuring that a COPD patient does not have a magnesium deficiency is clearly a crucial preventative step to take for keeping COPD under control, which would be highly beneficial if the patient were to contract COVID-19.

Flavonoids can be found in many fruits and vegetables and are known for their anti-oxidative and anti-inflammatory properties. These compounds have also been found to be central to a healthy respiratory system and especially beneficial in aiding the body in fighting respiratory tract infections.

### 3.5. Chronic Kidney Disease (CKD)

Chronic kidney disease is a progressive disease that causes the effectiveness of a patient’s kidney to decrease over time, resulting in a build-up of excess fluids and electrolytes in the body and can lead to kidney failure and death. The National Kidney Association recommends that people with CKD remain at home and avoid going out to crowded spaces as well as stocking up on shelf stable foods such as dried fruits, fruit juice, and canned fruits without added sugar [111]. However, according to the National Institute of Health, 468,000 people in the U.S. are on dialysis [112], making it challenging for them to remain isolated; thus, it would be beneficial to eat a kidney-friendly diet that boosts the immune system. In this section, we will discuss some of the challenges that people with CKD face as well as some general guidelines that may help improve their survivability chances concerning COVID-19.

#### Beneficial Nutrients and Minerals

Vitamin D is rarely found naturally in foods; rather, it can be found as an additive or dietary supplement but our primary source for vitamin D is its synthesis in our skin. All of these sources provide a biologically inert form of vitamin D, which is then processed in the liver to give us 25-hydroxyvitamin D and then in the kidneys to give us 1.25-dihyroxyvitamin D, which is the active form of vitamin D that plays a crucial role in many of our body’s functions, including the immune system [113]. As such, ensuring that patients with CKD do not have a vitamin D deficiency is crucial in the event of a COVID-19 infection as well as its benefit for a patient’s overall health. Due to the important role of the kidneys in producing the active form of vitamin D, it is important to ensure that all vitamin D produced is properly absorbed by the body. A recent literature review by the International Journal of Environmental Research and Public Health found that about 97% of people suffering from CKD and are on dialysis had vitamin D deficiency [113].

Although vegetables can be a source of vitamin D, people suffering from CKD should focus on absorption and activation since, as discussed, people with CKD may have issues converting it to its active form. Because vitamin D is fat-soluble, it is a reasonable conclusion to assume that fats can help with vitamin D absorption. However, a recent study found that not all fats are equal, as the study showed that long-chain triglycerides (in this study, peanut oil was used) produces higher vitamin D absorption results [114]. One well-known and readily available long-chain fatty acid is omega-3 polyunsaturated fatty acids.

Magnesium is also worth paying close attention to as this micronutrient is believed to lower the risk of death in patients with CKD, especially when accompanied by cardiovascular disease. This benefit is possibly due to magnesium’s protective effects against arterial calcification and hypertension [115]. This micronutrient may also help protect against phosphate toxicity; however, it is still inconclusive [116]. Magnesium is also important in activating vitamin D, which as discussed is crucial for people suffering from CKD [117].

Flavonoids are a group of compounds found in fruits and vegetables that have antioxidant and anti-inflammatory properties. These beneficial compounds have been found to have a protective effect for kidneys with people suffering from CKD [118]. These protective, anti-inflammatory, and anti-oxidative properties make flavonoid rich foods an essential part of healthy diet for people suffering from CKD, especially during the COVID-19 pandemic.

### 3.6. Hemoglobin Disorder Such as Sickle Cell Disease

Sickle cell disease (SCD) is caused by a group of hereditary blood disorders that affect the shape of the blood cells, making them curved, resembling a farm tool called a sickle. The disease causes symptoms that commonly include anemia, high risk of infection, and episodes of pain. SCD is fairly common in people with African descent with the CDC reporting that 1 in every 365 African-American births have SCD [119]. The CDC has found that SCD is one of the risk factors that can cause severe illness due to COVID-19 [5].

One of the symptoms of SCD is anemia due to early cell death. This form of anemia is not caused by iron deficiency rather it is simply a low number of living red blood cells causing many people who suffer from SCD to need blood transfusions making it more difficult for them to self-isolate. Another complication arising from the death of red blood cells and also combined with the curved shape of the cells is vaso-occlusive crises (VOC), which are caused by red blood cells dying and sticking to the walls of blood vessels, causing a blockage which in turn can lead to Acute Chest Syndrome (ACS) which can cause respiratory failure [120]. Considering how common this disease is, it is important to understand how COVID-19 affects people with SCD and what nutritional precautions they can take to help reduce the chances of severe complications, with an emphasis on boosting the immune system.

It has been observed that antioxidant compounds, especially flavonoids, found in fruits and vegetables can reduce the number of sickled cells [121]. Thus, an antioxidant-rich diet would be beneficial to people who suffer from SCD, both in reducing the severity of their symptoms and have the added benefit of supporting a healthy and robust immune system and a lower overall oxidative stress on the body. People suffering from SCD can incorporate FV in their diets to increase their flavonoid and antioxidant intake to help manage their symptoms and proactively boost their immunity to reduce the chances of serious illness if infected with COVID-19.

### 3.7. Immunocompromised State (Weakened Immune System)

During the COVID-19 pandemic, it is especially important for people with a compromised immune system to take proactive steps to help protect themselves. For the purpose of this article, we considered immunocompromised to mean all patients with a weakened immune system due to a medical condition or medication. Due to the wide range of reasons a person’s immune system may be suppressed, we cannot make direct connections between the condition and COVID-19 in regard to diet, so our focus will be on general dietary guidance for supporting the immune system.

#### Beneficial Nutrients and Minerals

Vitamin C is a strong antioxidant and an effective anti-inflammatory. It has been established that vitamin C plays a protective role in the respiratory system. The protective role of vitamin C makes it essential for people with a suppressed immunity even if their medications will keep their immunity suppressed to limit lung damage in case of infection with COVID-19. Recovery from lung damage caused by inflammation and pneumonia as a result of COVID-19 can, in some cases, take several months, and can cause long-lasting breathing issues.

Vitamin D plays an essential role in our immune response, so all deficiencies must be addressed during this pandemic. If a person has a compromised immune system due to medication taken after a solid organ transplant, vitamin D has the added benefit of decreasing the chances of organ rejection [122]. If someone with a compromised immune system has a deficiency it would be highly advantageous to ensure a high intake of magnesium as it helps activate vitamin D whether it was synthesized in the skin or absorbed from a dietary source [117].

Flavonoids are well studied, and their antioxidant and anti-inflammatory nature has been found to have organ protective properties [118], as well as being effective in aiding the body to fight off infectious respiratory diseases.

Since some nutrients and minerals have positive potential in managing symptoms of the chronic illnesses discussed above, as well their documented benefits to overall health, we compiled a list of fruits and vegetables that can be incorporated into a diet as a healthy and reliable source (Table 1).

## 4. Risks Associated with Quarantine and Lifestyle during COVID-19

A key feature of the COVID-19 pandemic has been mandatory shutdowns, stay-at-home orders, and self-isolation. While these precautions, in our opinion, have been necessary to slow the spread of COVID-19, it is important to address some of the issues that may arise from these measures. For example, many governments have shut down restaurants during this crisis, which in turn led many people to rely solely on home-prepared meals, which brings a host of potential risks with this abrupt change in eating habits. Another key issue to consider is the reduction in activity levels and sedentary habits that are a likely outcome of staying home which will also add to the effects of the dietary change. The combination of these two highly likely and outcomes of our lifestyle during the pandemic can increase the risks of developing unhealthy habits. These newly developed habits can cause harm to our mental and physical health, which, ironically, may increase our chances of severe illness or death if a person is infected with COVID-19. In this section, we will cover some of the issues we believe to be common and warrant special attention.

### 4.1. Risks of Relying Solely on Home Cooked Meals

Many people around the world rely on restaurants and food vendors for a significant portion of their diet. People may purchase their lunch from vendors at or around the workplace while others eat out as part of their recreation and socialization. This is especially apparent in industrialized countries as the CDC found that between 2013–2016, 36.3% of adults in the United States dined out on any given day [123]. As shutdowns affected restaurants in many countries and regions, people were forced to become more reliant on preparing their own meals, which in turn can potentially negatively affect some people’s diets in various ways. Some of the key dietary issues people should pay close attention to during shutdowns and stay at home orders are:

#### 4.1.1. Portion Sizes

With restaurants shutdown, people may attempt to cook some of their favorite meals that the usually would purchase at a restaurant. While this can be an enjoyable activity to learn new recipes, it is important to remember to pay close attention to the amount of food cooked to avoid over-eating and weight gain.

#### 4.1.2. Access to Fresh Fruits and Vegetables

The fast spread of COVID-19 resulted in supply chain disruptions as a result of closures. In the U.S. for example, a congressional research report issued in May 2020 found that the closures affected most farms as about half of U.S. farms rely on the food service industry to sell their produce. This change meant that a significant portion of the fresh fruits and vegetables grown were not available to the consumer as farms were not prepared to package their products for consumer sale, rather than bulk food service industry shipments, thus failing to recognize the risk of shortages [124]. Knowing of the stress recently experienced in the food chain, people should remember to stock up on fresh produce without over-shopping. It is also necessary to be mindful of the short shelf life of fresh produce and to eat what is purchased before it spoils to reduce waste. Recognizing the risk of shortages [124]. Knowing of the stress recently experienced by food chain, people should remember to stock up on fresh produce without over shopping. It is also necessary to be mindful of the short shelf life of fresh produce and to eat what is purchased before it spoils to reduce waste.

#### 4.1.3. Overconsumption of Canned Foods

While remaining at home during the pandemic, it may be appealing to depend on canned foods given their long shelf life. While the appeal is understandable, canned foods should not be heavily relied on due to additives found in them. Sodium is a major cause for concern with these products and according to the FDA sodium intake from packaged foods such as canned goods accounts for over 70% of American’s sodium intake [125]. With such high concentrations of sodium, it is easy to see how relying on canned goods can cause excessive sodium intake which can result in elevated blood pressure, CVD, and chronic kidney disease [126].

### 4.2. Inactivity

The most obvious outcome of staying at home during COVID-19 is that we tend to move less. This habit can lead to weight gain and obesity and all the associated health risks [127]. Lack of exercise can also weaken the immune system [128], so remaining active and healthy by using home exercises is beneficial for maintaining a healthy weight and overall health.

### 4.3. Depression

Depression is possible outcome of social isolation due to shutdowns and social distancing. Other stress factors such as loss of employment can also cause depression or make the depression worse. Depression in itself is a mental illness however it can also have physical manifestations such as a weakened immune system and cardiovascular disease [129]. Isolation induced depression may be alleviated with regular exercise [130]. Nutrition can also have an effect on depression as fried food, sweetened beverages, and high fat diets have been linked to depression while omega-3 polyunsaturated fatty acids has promising results in relieving depression symptoms [131]. So, exercise and a healthy diet can be beneficial to those feeling depressed while in isolation but also it may prove useful to utilize video call apps to contact friends and family and reduce the social isolation in a safe way.

### 4.4. Alcohol Abuse

People in isolation should pay close attention to the risk of alcoholism. The combination of isolation, boredom, and lack of employment may result in people over-indulging in alcohol and developing harmful drinking habits. In the U.S, alcohol sales rose considerably in March 2020, according to data from the National Institute of Health [132]. This rise is concerning as excessive consumption of alcohol can be detrimental to health. The CDC has found that in the United States, 22,246 people died as a result of alcoholic liver disease [133]. Alcohol affects the liver, and long-term alcohol abuse can cause kidney disease, immune dysfunction, and hypertension [134]. These damaging effects make excessive consumption of alcohol especially dangerous during the COVID-19 pandemic, as these conditions can increase the risk of severe illness or death if infected. Many governments have provided free resources to help people with unhealthy drinking habits that should be taken advantage of to address this problem.

### 4.5. Misinformation

As the COVID-19 pandemic spread around the world, conspiracy theories and false information started appearing on social media platforms and within social circles. The large amount of falsehoods and the quick pace spread led WHO to coin the phrase “infodemic” [135]. These rumors and conspiracy theories varied from harmless to potentially deadly as seen in the U.S. where an elderly couple self-medicated using chloroquine phosphate tablets, that are used for treating fish tanks, the couple did this after reading online about trials on hydroxychloroquine and believed that this may prevent the virus which resulted in the husband’s death [136]. These online rumors can also prevent the effectiveness of future measures to end the pandemic. [136]. These online rumors can also prevent the effectiveness of future measures to end the pandemic. A recent poll conducted in the United States found that 28% of Americans believe that Microsoft founder Bill Gates intends to implant microchips in people who take the COVID-19 vaccine [137]. This phenomenon emphasizes the need for the public to only get their information from public health organizations and local officials in order to make informed decisions regarding COVID-19.

## 5. Risk Associated with Food Handling

Consumption of fruits and vegetables is essential for a healthy life, as they are a great source of important components such as vitamins, minerals, and fibre [138]. Fruits and vegetables can be consumed in its raw form without any further processing. However, raw fruits and vegetables could lead to foodborne diseases [139]. In 2018, Food and Drug Administration (FDA) reported that, there is a huge number of outbreaks of food poisoning in United States caused by contaminated fruits and vegetable such as lettuce and spinach. There are many foodborne pathogens related to consumption fresh fruit and vegetables such as *Escherichia coli* O157:H7, Norovirus, Salmonella spp., Shigella spp., Hepatitis A virus, *Listeria monocytogenes*, and *Cyclospora cayetanensis*. Therefore, many food safety organizations stress the importance of washing fruits and vegetables before consumption [140]. It is necessary to ensure proper cleaning of fruits and vegetables and they are free from contamination especially during the ongoing pandemic.

Currently there is no evidence to prove that food is a source or route of transmission of COVID-19. However, coronavirus can survive on surfaces for various periods of time, depending on the material of the surface. It can exist for up to three days on plastic, and up to four days on stainless steel [141]. It can survive on food for one to three days [142]. It can survive on existing food packaging as well as fruits and vegetables handled by COVID-19-infected person [143]. It can remain on the surface of frozen food for up to three months [144]. Nevertheless, there is not any COVID-19 case identified because of consumption of contaminated food [145]. Even with food workers who handle food and have been infected with coronavirus, there is no evidence to prove that the virus can be passed to consumers via food or food packaging. The risk of transfer of coronavirus through food is by touching contaminated food and then touching one’s face [142].

According to the most recent data, it has been shown that coronavirus is not associated with food, but it remains necessary to follow hygiene recommendations to ensure and reduce any related risk. It is very important to follow safe hygiene practices, including handwashing. It is required to use water and soap for at least 20 s for hand washing. It must be cleaned hand after shopping, touching fresh food or food packaging, and before preparing or eating food. If soap and water is not available, the use of hand sanitizer containing at least 60% alcohol should be properly used [5].

The CDC and WHO have suggested practices to ensure cleaning of fruits and vegetables before consumption [5,145]. The best practice is washing fruits and vegetables thoroughly before consumption [142]. It is recommended to wash hands first, then rinse fruits or vegetables under running water without soap, and wash hands afterwards [146]. WHO suggested to use potable water in cleaning as COVID-19 has not been found in drinking water [142].

There may be difficulty in cleaning some types of vegetables, such as mushrooms, because of their ability to absorb water. Therefore, it is recommended to use a colander and wash them. Afterwards, they can be dried by using a clean kitchen towel or paper towels. Further types of food that could be difficult to clean are soft vegetables and fruits, such as ripe fruit, berries and tomatoes. To avoid crushing and loss of the product, it is advised to rinse them under water with low pressure, then put the product on a clean kitchen towel or paper towels to dry [147]. Moreover, it is necessary to regularly clean and sanitize surfaces in the kitchen before preparing food [5].

## 6. Conclusions

Because of the novel nature of COVID-19, research is still ongoing. In this paper, we explored some of the preexisting conditions that increase the likelihood of severe illness and used previously available research to determine ways of managing some of the symptoms that appear to be linked to COVID-19 hospitalizations. This article presents many micronutrients and minerals, especially Vitamin C, Vitamin D, and flavonoids, which can be used as a proactive dietary supplement to help manage symptoms and to reduce the risk of severe illness from COVID-19. We found that many of the risk group diseases shared common symptoms that are linked to severe illnesses from COVID-19 further supporting that nutrition is relevant to this virus however more epidemiological research is necessary to identify the supportive role nutrition may have in the protection of at-risk groups. These dietary suggestions have been based on what we know about COVID-19 as well as on our knowledge of how nutrition interacts with our immune system and chronic conditions however personal dietary requirements vary considerably so patients must always consult their primary care physician for specific guidelines suited to their needs. Furthermore, research in the role nutrition plays is extensive however, further studies are needed in the relation between micronutrient and its mechanism on how it effects COVID-19 patients. Furthermore, the role of cytokine storms and how they may relate to nutrition is an area with significant research gaps that we believe to be highly beneficial. We also believe that research in nutrition epidemiology for patients who were hospitalized is warranted. A primary area of focus in the epidemiological research should focus on deficiencies that people hospitalized may have had during COVID-19 era.

## Figures and Tables

**Table 1:** Fruits and vegetables, its important nutrients and minerals related to COVID-19.

Fruit/Vegetable	Nutrient/Mineral	Patients That May Benefit	References
Red beets	Contain Folate, fiber, copper, magnesium, and potassium	CVD & Diabetes	[27,28]
Squash (e.g., acorn & pumpkin)	Vitamins A, B1, B6, C, calcium, carotenoids, copper, fiber, folate, magnesium, manganese, and potassium	Diabetes, CVD, Obesity, COPD, compromised immune system, CKD.	[27,29]
Apples & Pears	Vitamin C, fiber, and flavonoids	CVD, Obesity, COPD, Compromised Immune System, CKD	[27,30,31]
Berries (e.g., raspberries blue berries and black berries)	“Vitamin C, folate, manganese, potassium, fiber” and flavonoids	CVD, Obesity, Diabetes, compromised immune system	[27,32]
Mangoes	“Vitamins A, C, E, potassium, and fiber”	CVD, Obesity, Diabetes, compromised immune system	[27]
Grapes	Contain high levels of polyphenols and Flavonoids	SCD, COPD, CKD, compromised immune system.	[27,32,33]
Soybeans	A good source of Magnesium, calcium and omega-3 polyunsaturated fatty acids	COPD, CKD, CVD, Diabetes, Compromised immune system.	[34,35,36,37]
Oranges	Rich in flavonoids, Vitamin C	SCD, COPD, CKD, CVD, Obesity, Diabetes, Compromised immune system.	[38,39,40]
Parsley	Rich in flavonoids	SCD, COPD, CKD, Compromised immune system.	[41]
Peppers (bell peppers and chili peppers)	A good source of antioxidants such as flavonoids and Vitamin C	SCD, COPD, CKD, Obesity, CVD, Diabetes, Compromised immune system.	[38,42,43,44]
Broccoli	Rich in antioxidants such as flavonoids and Vitamin C	SCD, COPD, CKD, Obesity, CVD, Diabetes, Compromised immune system.	[38,45,46]
Onions	Has a wide variety of flavonoids	SCD, COPD, CKD, Obesity, Compromised immune system.	[47,48]
Spinach	A good source of antioxidants, Vitamin C, iron, magnesium	CVD, Obesity, Diabetes, COPD, CKD, Compromised immune system.	[34,38,49]
White & Kidney Beans	A good source of iron, potassium and magnesium	COPD, CVD, Diabetes, CKD, Compromised Immune system.	[34,49,50,51]
Bananas	A good source of magnesium and potassium	CVD, COPD, CKD, Diabetes, Compromised immune system.	[34,50]
Cantalope	A great source for Vitamin C	CVD, Obesity, Diabetes, COPD, Compromised immune system.	[38]
Mushrooms	A good source of dietary Vitamin D	Diabetes, CKD.	[52]
